# Heterogeneous Patterns of Wearing-Off in Parkinson’s Disease

**DOI:** 10.3390/jpm16100518

**Published:** 2026-10-03

**Authors:** Gabriella K. Guerra, Shermyn Neo, Maryam S. Mirian, Sepideh Hajipour Sardouie, Emad Arasteh, Martin Keung, Christina Kim, Juana Ayala Castaneda, Somdattaa Ray, Martin J. McKeown

**Affiliations:** 1 Pacific Parkinson’s Research Centre, University of British Columbia, Vancouver, BC V6E 2M6, Canada; gabriella.guerra@ubc.ca (G.K.G.); shermyn.neo.x.m@singhealth.com.sg (S.N.); maryam.mirian@ubc.ca (M.S.M.); sepideh.hajipour@ubc.ca (S.H.S.); emad.arasteh@ubc.ca (E.A.); martin.keung@ubc.ca (M.K.); christina.kim@ubc.ca (C.K.); juana.ayala@ubc.ca (J.A.C.); 2 National Neuroscience Institute, Singapore 308433, Singapore; 3 Department of Neurology, St. Johns Medical College Hospital, Bangalore 560034, India; somdattaa.ray@stjohns.in

**Keywords:** Parkinson’s disease, wearing off, ecological momentary assessment (EMA), WOQ-19, symptom heterogeneity, levodopa, digital health

## Abstract

**Background:** Wearing-off (WO) in Parkinson’s disease (PD) refers to the recurrence of motor and non-motor symptoms between levodopa doses. Its prevalence, inter-individual variability, and temporal relationship to medication remain incompletely characterized in real-world settings. **Methods:** Twenty-three PD participants with self-reported WO completed a digitized Wearing-Off Questionnaire-19 (WOQ-19) hourly during waking hours over three consecutive days (72 h), yielding 894 valid surveys. Symptom heterogeneity was quantified using the coefficient of variation (CV) and intraclass correlation coefficient (ICC). Symptom changes time-locked to medication intake were assessed using Cochran’s Q test. **Results:** Mean compliance was 91.86% ± 6.31%. All 19 WOQ-19 symptoms were endorsed at least once, with participants reporting a median of 8 distinct symptoms (IQR: 5–11). Prevalence differed significantly across symptoms (Kruskal–Wallis test, *p* < 0.001). Tremor was the most prevalent symptom overall, while other symptoms were rare, episodic, and highly variable across individuals. Participants’ relative symptom burden was consistent between Days 2 and 3 (Spearman’s rho = 0.682, *p* < 0.001), and diurnal worsening showed a non-significant positive trend (β = 0.031/hour, *p* = 0.117). Five symptoms showed significant peri-dose modulation: weakness, muscle cramping, experience of hot or cold, tremor, and abdominal discomfort (all *p* < 0.05). WO burden did not significantly correlate with standard clinical severity measures. **Conclusions:** WO symptom expression is highly heterogeneous across individuals but largely consistent from day to day within individuals. Weakness and experience of hot or cold are candidates for future wearable sensor-based studies of levodopa cycling, supporting individualized approaches to WO assessment in PD.

## 1. Introduction

Parkinson’s disease (PD) is a progressive neurodegenerative disorder characterized by motor features such as bradykinesia, rest tremor, and rigidity, as well as a broad spectrum of non-motor symptoms [1]. Levodopa remains the most effective pharmacological therapy for PD motor symptoms. However, as the disease progresses and central dopaminergic reserve diminishes, many patients develop motor and non-motor fluctuations. The most common of these is wearing off (WO): a generally predictable recurrence of motor or non-motor symptoms that precedes a scheduled dose and typically improves with anti-parkinsonian medication [2].

WO affects approximately 40–50% of people with PD (PwP) within the first five years of levodopa therapy, rising to over 70% after ten years [3]. WO has substantial impact on quality of life, functional independence, and activities of daily living [4]. Despite this, WO remains underdiagnosed and incompletely characterized in routine clinical practice [5]. Traditional clinical assessment through episodic clinic visits, cross-sectional rating scales, and brief ON/OFF testing in controlled settings, captures only a limited picture of the patient experience. These approaches are susceptible to recall bias, visit-to-visit variability, and limited relation to real-world symptom fluctuations [6].

The WOQ-19 is a validated patient-reported instrument that enumerates 19 motor, autonomic, sensory, and neuropsychological symptoms commonly associated with WO [7]. While the WOQ-19 has been used as a screening and diagnostic tool, it has rarely been administered at high temporal resolution over multiple days in naturalistic settings. Most existing digital symptom studies in PD are short in duration, focus on a narrow range of motor outcomes, or lack systematic medication timing data [8,9]. Consequently, the full portrait of WO symptom expression, including inter-individual variation, diurnal evolution and levodopa sensitivity, remains incompletely described.

Applying Ecological Momentary Assessment (EMA), we used a custom iOS mobile application (see Section 2.2) to capture hourly WOQ-19 responses over 72 consecutive hours, alongside real-time medication logging, to characterize the prevalence, inter-individual heterogeneity, temporal dynamics, and medication sensitivity of all 19 WO symptoms in a cohort of community-dwelling adults with PD.

## 2. Methods

### 2.1. Participants

Participants who met the following inclusion criteria were included: idiopathic PD diagnosed by a certified movement disorder specialist, age 18–85 years, self-reported experience of at least 2–3 WO episodes per day for the past month, familiarity with smartphone use, and fluency in English. Participants were excluded if they had: atypical parkinsonism, depression (Beck’s Depression Inventory-II > 14), cognitive impairment (Montreal Cognitive Assessment score ≤ 21), history of deep brain stimulation, pacemaker or any other device sensitive to small electrical signals, anticholinergic medication use or any condition that may affect the autonomic nervous system.

### 2.2. Study Design

At a baseline study visit, participants were trained to use a custom iOS mobile application (developed in collaboration with the UBC Cloud Innovation Centre (CIC), University of British Columbia, Vancouver, BC, Canada) containing a digitized version of the WOQ-19. They were instructed to complete the digital WOQ-19 hourly during a 12 h daily activity window for three consecutive days (72 h total), yielding 13 surveys per day. The standard daily activity window was 09:00–21:00 but two participants opted for 07:00–19:00. Hourly alarms were set on the mobile phone as a reminder to complete each questionnaire. Medication logging was performed in the same application throughout the designated window. Participants adhered to their usual medication dosing schedule without modification. All analyzed participants were taking levodopa/carbidopa; peri-dose analyses used logged dose timestamps as levodopa-aligned dosing events (the app records timing rather than drug identity).

We collected demographic information (age, sex, disease duration), and administered the Beck Anxiety Inventory (BAI) and the Scale for Outcomes in Parkinson’s Disease—Autonomic Dysfunction (SCOPA-AUT). The Movement Disorders Society–Unified Parkinson’s Disease Rating Scale Part III (MDS-UPDRS-III) was used to gauge motor severity.

The WOQ-19 captures 19 symptoms across four domains: motor, autonomic, neuropsychological and sensory. While the original WOQ-19 has a second column for patients to indicate if symptoms improve with medication, this was omitted in the digital WOQ-19 for two reasons: firstly, many users misunderstand how to complete the questionnaire and may check only one column, creating uncertainty about whether they actually experience a WO effect or a random symptom [10,11]. Secondly, PwP often have difficulty differentiating ON–OFF states and appreciating medication responsiveness of symptoms. We therefore asked participants to focus on the presence or absence of symptoms alone, and in the primary analysis we correlated responses with medication timing for an objective interpretation of WO symptoms.

### 2.3. Data Processing

Questionnaire entries were time-rounded to the nearest scheduled hour within ±20 min. Questionnaires completed outside the daily 12 h window or more than 20 min from a scheduled timepoint were excluded. In cases where more than one questionnaire was completed at the same timepoint, only the most recently submitted entry was retained. Medication entries timestamped within 30 min of another entry were collapsed into a single dosing event to account for participants who logged individual pills rather than doses. Overnight periods were excluded from inter-dose interval (IDI) calculations. Within-day consecutive dose pairs used for IDI and IDI-normalised analyses are defined in Section 2.4.

Participants who completed fewer than 30 of the 39 expected questionnaires (<75% compliance) were excluded from analysis, as pre-specified, to preserve data completeness and consistency. Compliance was assessed within each participant’s 72 h protocol window (beginning 1.5 h after the baseline visit); for retained participants, all valid questionnaires from study days 1–4 were analyzed.

### 2.4. Statistical Analysis

Symptom prevalence was defined as the proportion of completed surveys per participant in which a given symptom was positively endorsed, expressed as a percentage. A Kruskal–Wallis test was used to assess overall differences in symptom prevalence distributions across the 19 symptoms.

Inter-individual heterogeneity was characterized by the coefficient of variation (CV = standard deviation/mean × 100%) computed across participant-level prevalence values for each symptom. CV reflects the degree to which a symptom is expressed variably across the cohort, with high CV indicating more heterogeneity. Since the data was binary, prevalence directly affects CV in a non-linear fashion, with near-zero CV at high prevalence and vice versa.

Within- versus between-individual variance was partitioned using the intraclass correlation coefficient (ICC = between-person variance/total variance). High ICC indicates that individuals differ from each other in a stable, trait-like manner while low ICC indicates that the symptom is state-like or fluctuating within individuals over time. The ICC–CV plane was divided into four interpretable quadrants using a threshold of 0.5 for ICC to reflect sufficient within-person variance relative to between-person differences. The median of the observed CV distribution was used as an arbitrary split threshold, as there is no established prevalence threshold for symptoms. Instead, other considerations like the consequence of missing a symptom, and the actionability and reliability of symptom monitoring are more important (see Discussion Section).

Dose-locked symptom trajectories were constructed by computing each participant’s time since the most recent levodopa dose (τ) for each survey. For IDI calculations and IDI-normalized trajectories, within-day consecutive dose pairs were defined after collapsing medication entries logged within 30 min into a single dosing event: successive collapsed doses occurring on the same calendar day formed pairs (e.g., four doses in one day yield three pairs: 1–2, 2–3, and 3–4). The final dose of each day was not paired (no within-day subsequent dose). Overnight intervals and near-zero gaps (<0.5 h) were excluded. These pairs were not treated as independent observations: consecutive pairs from the same participant share dosing events and abut in time, and multiple pairs arise from the same individual. For the IDI-normalized dose-locked trajectory, surveys within each pair interval were used to describe the cycle, but cohort summaries were obtained by averaging within participants first and then across participants, so individuals contributed equally regardless of how many pairs they contributed. Cochran’s Q analysis tested whether the probability of endorsing a symptom changed significantly across time bins relative to a medication dose; the analysis matrix was organized by dose events nested within participants, and the permutation null reshuffled bin labels within participant blocks, thereby preserving within-person dependence.

Also, Cochran’s Q used a fixed −2 to +2 h window around each dose, with surveys assigned to hourly bins (1 h windows centred on each bin); a dose contributed only if all five bins were observed (complete-case rows; *n* = 122). When the within-day inter-dose interval was <4 h—which is common in this cohort (median IDI ≈ 3 h)—these windows could overlap in absolute time with the peri-dose period of the adjacent dose, and a given survey could be mapped into more than one dose-centred row (with different relative-time labels). Windows were not truncated at the subsequent dose, nor was exclusive nearest-dose assignment enforced. Inference used participant-blocked permutations, which partly address within-person dependence arising from such overlap. As a complementary approach that avoids fixed-hour overlap by design, IDI-normalized dose-locked trajectories restricted observations to the interval between consecutive within-day doses. Formal exclusion of overlapping Cochran windows (e.g., IDI-capped bins or exclusive dose assignment) was not applied.

Diurnal and day-to-day patterns were examined using a linear mixed-effects (LME) model with WOQ-19 total score as the outcome, time of day and study day as fixed effects, and participant as a random intercept. Partial days were excluded from this analysis. Per-participant mean symptom prevalence on Days 2 and 3 were compared using Spearman to assess day-to-day consistency of symptom burden.

Spearman correlations were computed between each participant’s mean WOQ-19 total score and five pre-specified clinical variables to evaluate clinical correlates of symptom burden. Ninety-five percent confidence intervals for Spearman rho were obtained by percentile bootstrap (10,000 resamples).

Analyses were performed in MATLAB R2024b (The MathWorks, Inc., Natick, MA, USA) and Python 3.12 (Python Software Foundation, Wilmington, DE, USA).

## 3. Results

### 3.1. Feasibility and Adherence

Of 30 recruited participants, 7 were excluded for completing fewer than 75% of expected questionnaires. Of 1378 submitted questionnaires, those submitted outside the daily 12 h window (*n* = 140), more than 20 min from a scheduled timepoint (*n* = 64), duplicating a timepoint (*n* = 61; only the most recent was retained), or outside study days 1–4 (*n* = 34) were removed, and questionnaires from excluded participants (*n* = 185) were set aside, leaving 894 valid surveys from 23 participants. Detailed comparisons of included (*n* = 23) and excluded (*n* = 7) participants are provided in Supplementary Table S1. The groups did not differ significantly on age, sex, disease duration, MoCA, BDI-II, BAI, MDS-UPDRS-III motor severity, Hoehn & Yahr stage, or self-reported WO episode frequency. The only significant difference was a higher SCOPA-AUT score in the excluded group (16.43 ± 4.61 vs. 12.18 ± 2.87; *p* = 0.018); in isolation, greater autonomic symptom burden is not expected to meaningfully alter the core wearing-off phenomenon under study. Both groups were similar in disease duration and clinical motor severity based on MDS-UPDRS-III.

The mean completion rate across the retained cohort was 91.86% ± 6.31%, corresponding to approximately 36 completed surveys per participant across the 72 h study period. The unsigned mean reporting delay from the hourly reminder alarm was 2.20 ± 3.77 min. A chi-square goodness-of-fit test confirmed that survey completion was uniformly distributed across hours of the day with no significant association between time of day and missingness (χ^2^(12) = 4.78, *p* = 0.965).

### 3.2. Participant Cohort Characteristics

Demographic and clinical characteristics of the final cohort are shown in Table 1. The mean age was 66.26 ± 8.47 years with a mean disease duration of 9.61 ± 5.48 years. By Hoehn & Yahr stage, 6 participants (26.1%) were HY1, 12 (52.2%) were HY2, and 5 (21.7%) were HY3. Participants self-reported a range of 1–8 WO episodes per day. The cohort median was 2.5 WO episodes per day (IQR: 2–3), with a mean of 2.87 ± 1.42.

### 3.3. Medication Summary

After collapsing 42 dose entries timestamped within 30 min of one another, participants reported a median of 5 doses per full day (IQR: 4–5.5). The mean inter-dose interval was 3.30 ± 1.36 h, while the median inter-dose interval was 3.03 h (IQR: 2.50–3.96).

All 23 participants in the analyzed cohort were prescribed levodopa/carbidopa and adhered to their usual oral dosing schedule during monitoring. Medication events used in peri-dose analyses were participant-logged dose times; the mobile log records timing rather than drug identity, and peri-dose analyses were framed around these levodopa-aligned dosing events. In this cohort, levodopa/carbidopa constituted the primary dopaminergic regimen for all participants. Baseline records identified concomitant dopaminergic therapies: entacapone (*n* = 3), MAO-B inhibitors (rasagiline, *n* = 1; selegiline, *n* = 1), and dopamine agonist therapy (rotigotine, *n* = 2). Altogether, 7/23 participants used at least one adjunct. These agents can modify levodopa pharmacokinetics and pharmacodynamics and may influence the timing or magnitude of peri-dose symptom trajectories; given the small subgroup sizes, formal stratified sensitivity analyses were not performed.

### 3.4. Symptom Prevalence

All 19 symptoms on the WOQ-19 were endorsed at least once across the cohort. Participants reported experiencing between 2 and 16 distinct symptoms over the study period, with a median of 8 distinct symptoms (IQR: 5–11).

**Figure 1:**
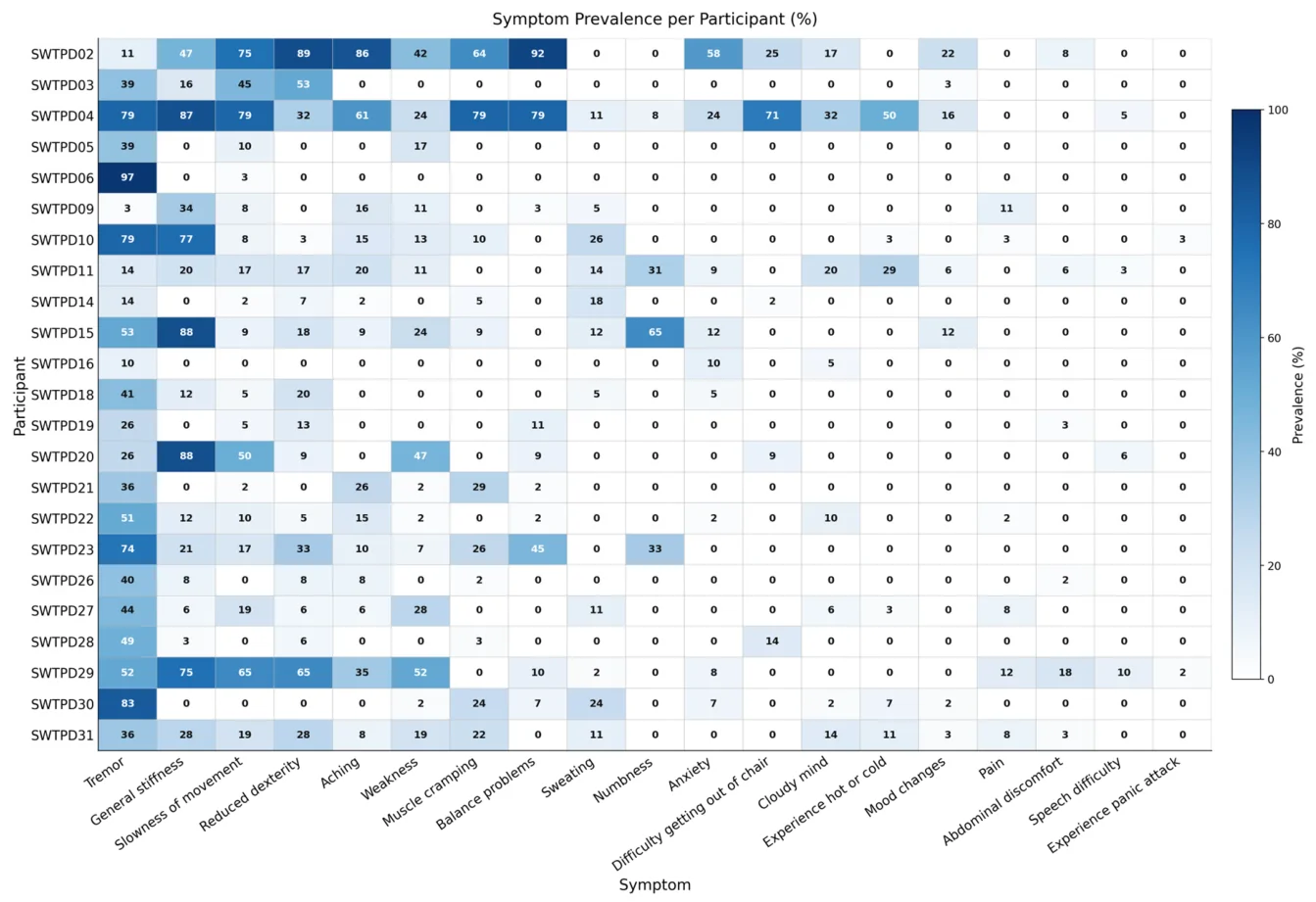
Participant x symptom prevalence heatmap.

Participant-level symptom prevalence—the proportion of a participant’s completed surveys in which each symptom was endorsed—revealed pronounced heterogeneity across the cohort (Figure 1).

Participant × symptom prevalence heatmap. Each cell shows the percentage of completed hourly surveys in which a given symptom was positively endorsed by a given participant. Symptoms are ordered left-to-right by descending cohort mean prevalence. Colour scale: white = 0%, dark blue = 100%. A Kruskal–Wallis test confirmed that the distribution of prevalence values differed significantly across symptoms (χ^2^(18) = 131.98, *p* = 2.23 × 10^−19^). Thirteen of the 19 symptoms (68.4%) exhibited strong zero-inflation, with more than half of participants reporting absence of that symptom.

Figure 1 summarizes participant-level symptom prevalence across the cohort (ordered by descending mean endorsement frequency). Mean symptom frequencies across patients are shown in Supplementary Figure S1. Motor symptoms were most prevalent overall, with tremor the most commonly endorsed symptom (mean patient-level prevalence 43.4%), though still below half of awake hours on average. General stiffness, slowness of movement, and reduced dexterity ranked next; aching and weakness were the most prevalent sensory symptoms. Among autonomic symptoms, sweating was the most frequently endorsed (6.1% mean prevalence).

### 3.5. Symptom Heterogeneity—ICC-CV Quadrant Framework

**Figure 2:**
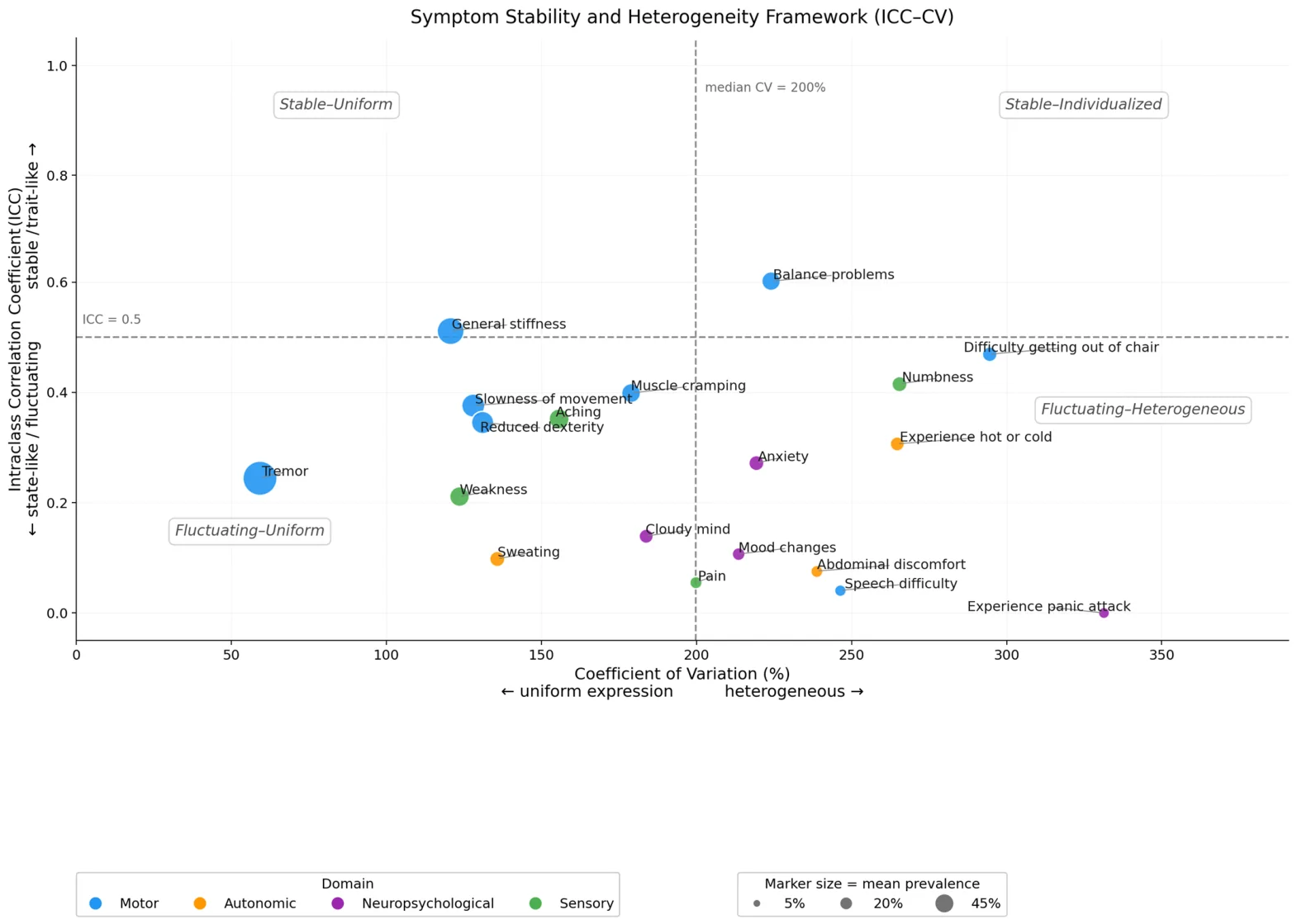
ICC-CV quadrant framework. ICC–CV quadrant framework for wearing-off (WO) symptom characterization. X-axis: coefficient of variation (CV; inter-participant heterogeneity of patient-level prevalence). Y-axis: intraclass correlation coefficient (ICC; between-person variance/total variance; high = trait-like/stable within person, low = state-like/fluctuating). Dashed lines divide the space at ICC = 0.5 and the cohort median CV into four quadrants. Marker size is proportional to mean patient-level prevalence (see size legend).

Plotting ICC against CV for each symptom (Figure 2) separated symptoms into four interpretable quadrants. As noted under Methods, CV is an inverse reflection of prevalence as the data was binary.

Stable–Uniform profile (high ICC, low CV): General stiffness only. This symptom shows the strongest within-person stability relative to between-person differences, with comparatively modest inter-participant heterogeneity in mean endorsement frequency.

Stable–Individualized profile (high ICC, high CV): Balance problems. This symptom is relatively stable within individuals over time but varies substantially in level across the cohort.

Fluctuating–Uniform profile (low ICC, low CV): Tremor, slowness of movement, reduced dexterity, aching, weakness, muscle cramping, sweating, and cloudy mind. These symptoms are broadly shared across participants (several with moderate mean prevalence) but fluctuate within individuals over time—i.e., state-like rather than trait-like. Tremor has the highest mean prevalence and the lowest CV in this group.

Fluctuating–Heterogeneous profile (low ICC, high CV): Difficulty getting out of a chair, numbness, experience of hot or cold, anxiety, mood changes, abdominal discomfort, pain, speech difficulty, and experience of panic attack. These symptoms are episodic and highly variable across the cohort; many show zero-inflated patient-level distributions (median prevalence often 0%).

This quadrant framework provides a practical taxonomy: from symptoms with relatively stable, cohort-uniform expression (general stiffness), to stable but individualized burden (balance problems), to broadly shared but time-varying symptoms (tremor, slowness, weakness, muscle cramping), and finally to rare, episodic phenomena (mood changes, speech difficulty, panic attack). Symptoms near quadrant boundaries (e.g., general stiffness at ICC ≈ 0.51; difficulty out of chair, numbness, and hot/cold just below ICC = 0.5) should be interpreted as approximate groupings rather than rigid classifications.

### 3.6. Medication Dose-Locked Symptom Trajectories

The cohort symptom trajectory showed the expected pharmacodynamic pattern: symptom burden was highest at the beginning of the cycle, reflecting peak wearing-off at the time of dosing, decreased to its minimum around 25–45% of the cycle as levodopa took effect, and rose again toward the next dose, consistent with declining plasma levodopa levels. Substantial inter-individual variability was observed in the magnitude and shape of this trajectory, reflecting differences in disease stage, medication regimen, and individual levodopa pharmacokinetics (Supplementary Figure S2).

**Figure 3:**
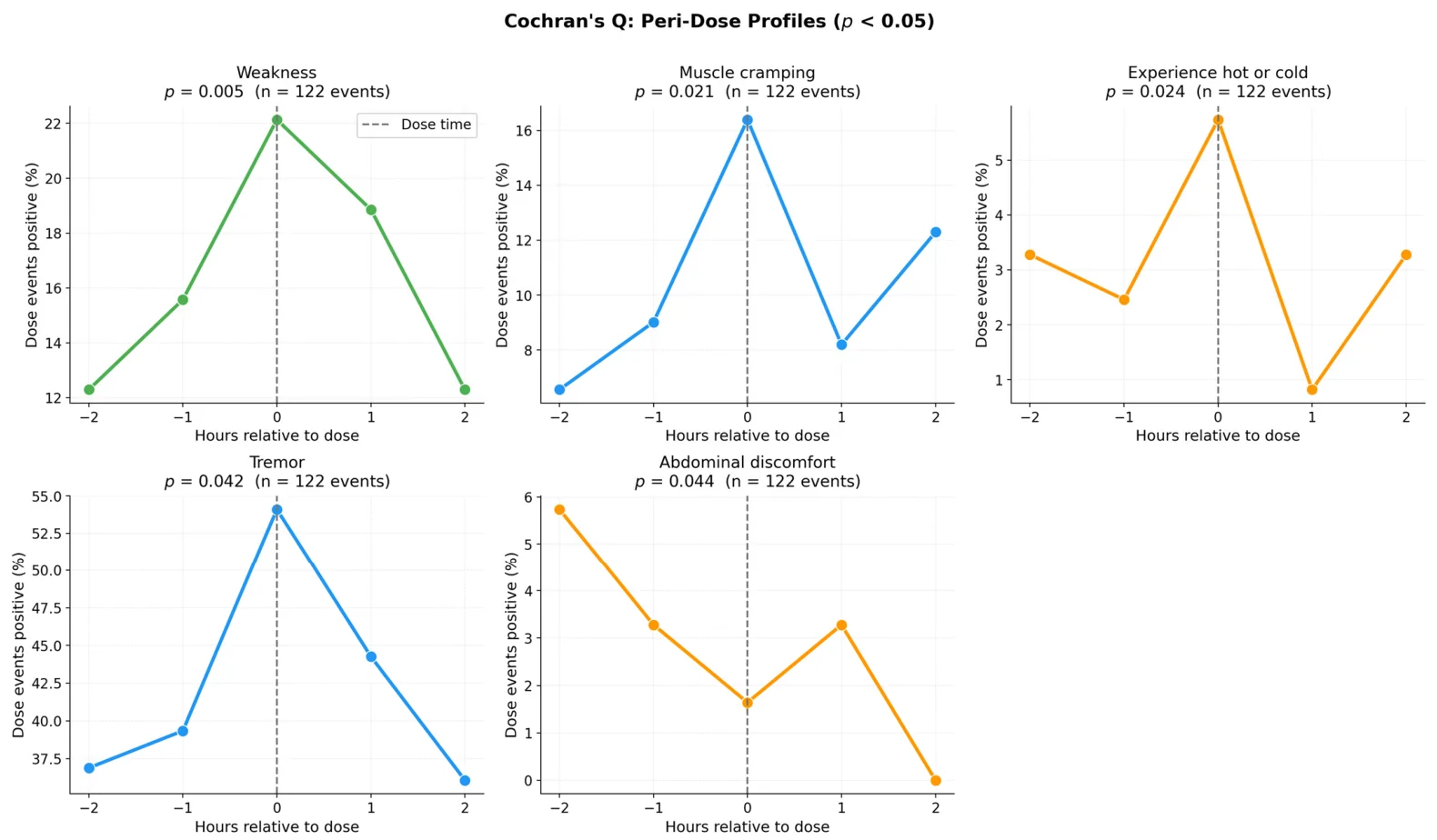
Cochran’s Q peri-dose profiles. Cochran’s Q peri-dose profiles for the five symptoms with significant temporal modulation around levodopa dosing (permutation *p* < 0.05; *n* = 122 complete dose events per symptom). X-axis: hours relative to dose (−2 to +2). Y-axis: percentage of dose events with a positive symptom endorsement in each hourly bin. Vertical dashed line marks dose time. Symptoms shown (ascending *p*): weakness (*p* = 0.005), muscle cramping (*p* = 0.021), experience of hot or cold (*p* = 0.024), tremor (*p* = 0.042), and abdominal discomfort (*p* = 0.044).

Cochran’s Q analysis. To assess symptom improvement with medication, Cochran’s Q analysis was applied to examine the probability of endorsing each symptom across hourly time bins in the −2 to +2 h peri-dose window. A total of 122 complete dose events (across all 23 participants) contributed to the analysis matrix. Five symptoms reached statistical significance after permutation testing (*p* < 0.05; Figure 3): weakness (Q = 8.57, *p* = 0.005), muscle cramping (Q = 13.55, *p* = 0.021), experience of hot or cold (Q = 7.83, *p* = 0.024), tremor (Q = 14.54, *p* = 0.042), and abdominal discomfort (Q = 9.71, *p* = 0.044). Slowness of movement showed a marginal trend (Q = 8.22, *p* = 0.051).

### 3.7. Diurnal and Day-to-Day Patterns

A linear mixed-effects model of WOQ-19 total score across time of day (restricted to full days, i.e., Days 2 and 3) showed a positive trend for diurnal worsening—evening scores (18:00–21:00) were on average 0.23 points higher than morning scores (09:00–12:00)—however this did not reach statistical significance (β = 0.031 per hour, *p* = 0.117; Supplementary Figure S3). There was no significant main effect of study day (*p* = 0.245) and no significant time-of-day × study-day interaction (*p* = 0.059), indicating that the diurnal pattern was consistent across study days.

**Figure 4:**
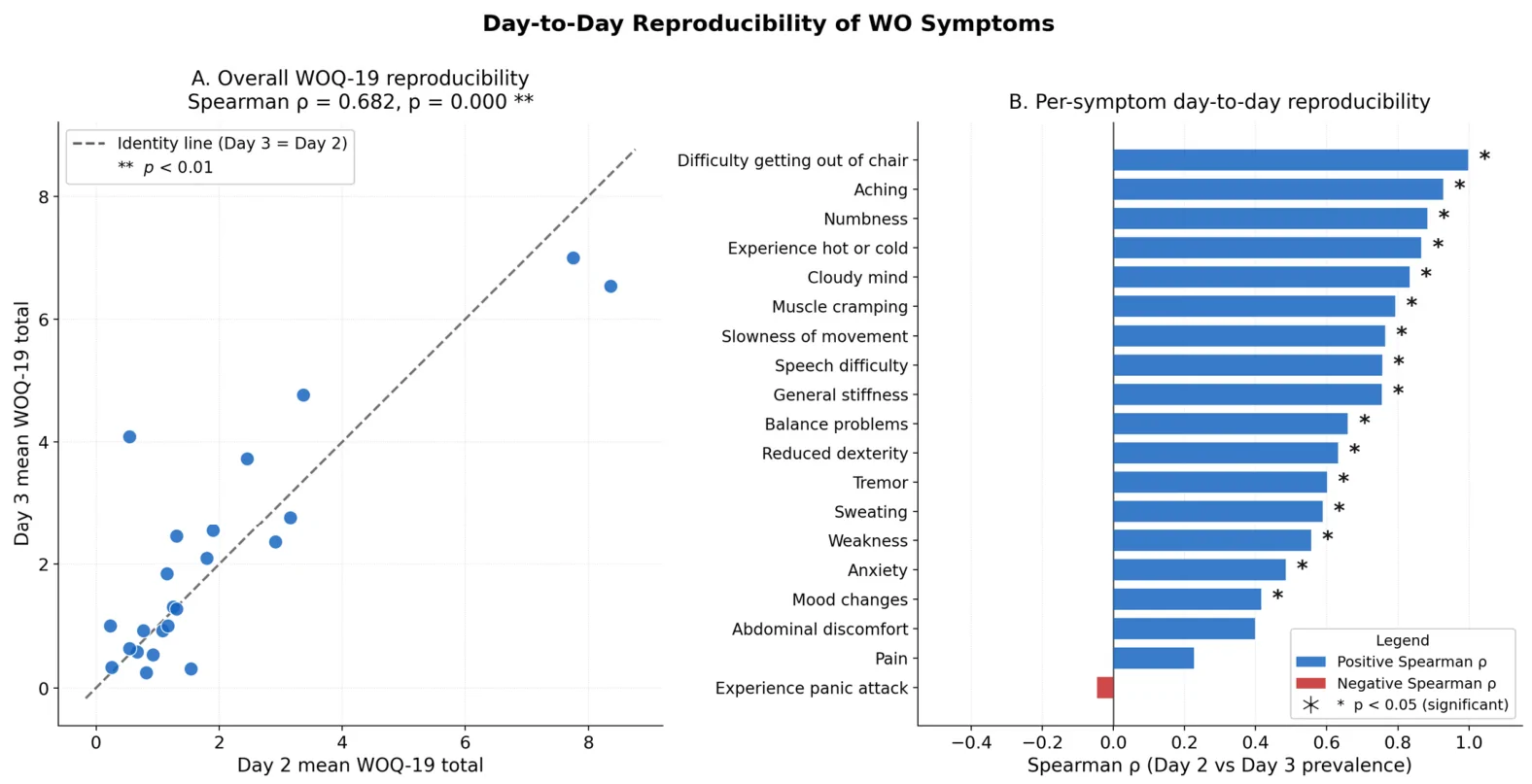
Day-to-day Consistency. Each point is one participant’s mean WOQ-19 total on Day 2 versus Day 3 (*n* = 23). The dashed line is the identity line (Day 3 = Day 2).

Day-to-Day Consistency. This was assessed by comparing per-participant mean symptom prevalence between Day 2 and Day 3. Overall WOQ-19 total scores were strongly correlated between days (Spearman rho = 0.682, *p* < 0.001; Figure 4A), indicating that the relative symptom burden of individual participants within the cohort remained stable across consecutive days.

At the symptom level, 16 of 19 symptoms showed statistically significant Day 2–Day 3 correlations (*p* < 0.05; Figure 4B).

These results suggest that symptom patterns are largely consistent across consecutive days, supporting the use of multi-day EMA for individual profiling; however, formal test–retest reliability remains to be established. Symptoms with the poorest day-to-day consistency fell in the rare, episodic, and heterogeneous quadrant in the ICC–CV analysis (low ICC, high CV), providing convergent evidence that these symptoms are genuinely intermittent.

Day-to-day consistency of WO symptoms. (A) Scatter of per-participant mean WOQ-19 total on Day 2 (x-axis) vs. Day 3 (y-axis). The dashed line is the identity line (perfect day-to-day agreement: Day 3 = Day 2), not a regression fit. Spearman rho and *p*-value are shown in the panel title. (B) Spearman rho for Day 2 vs. Day 3 per-participant symptom prevalence.

### 3.8. Clinical Correlates of Symptom Burden

Spearman correlations between each participant’s mean WOQ-19 total score and five pre-specified clinical variables are reported in Table 2 (with 95% bootstrap confidence intervals). No association reached statistical significance. Point estimates were small-to-moderate in magnitude (rho ranging from −0.25 to +0.26), and all 95% CIs included zero. Given the limited sample size (*n* = 23), these non-significant findings should be interpreted cautiously and do not, by themselves, establish a true dissociation between EMA-derived burden and clinic-based ratings. Rather, they are consistent with the possibility that EMA and conventional assessments capture complementary aspects of disease (day-to-day lived symptom burden versus cross-sectional clinical severity).

## 4. Discussion

This study provides a detailed characterization of WO symptom experience in PD, using hourly self-report over three consecutive naturalistic days. We found that WO symptom expression (1) is highly heterogeneous across individuals, (2) is highly consistent day to day within individuals, and (3) does not correlate significantly with standard clinical severity measures, highlighting the value of Ecological Momentary Assessments (EMAs). An ideal EMA biomarker requires convergence of several features: reliable improvement with medication (actionability), low ICC (state-like property), and day-to-day consistent. While a low CV (high prevalence) is preferable, we note that WO symptoms are highly individualized and EMA likely needs to be tailored.

### 4.1. Heterogeneity as a Core Feature of WO

The pronounced inter-individual heterogeneity in symptom expression that we observe reflects a fundamental characteristic of WO in PD familiar to most experienced clinicians: namely, there is no single, canonical symptom profile when dopaminergic medication wears off. While tremor was the most common symptom and was reported at least once by every participant, it was endorsed in fewer than half of hourly surveys on average (mean prevalence 43.4%), and its frequency ranged widely across individuals (3–97% of surveys). This finding reinforces the inadequacy of group-level symptom profiles for characterizing individual patient burden and has direct clinical implications: WO recognition and monitoring strategies must be tailored to the individual. Self-report tools like the WOQ-19, which enumerate the full symptom space, are better suited to individual profiling than global ON/OFF ratings, which lack symptom-level resolution.

### 4.2. The Ideal Biomarker of WO

The ICC–CV framework we employed offers a novel and clinically interpretable taxonomy of WO symptom types. Symptoms falling in the Fluctuating–Uniform quadrant (low ICC, low CV) are broadly shared across the cohort but exhibit time-varying, state-like behaviour within individuals, making them strong candidates for continuous sensor-based monitoring of within-person fluctuations. Of these symptoms, tremor has the most ideal profile and is an established monitoring biomarker for WO, as with slowness of movement (bradykinesia) [12,13]. While many symptoms, such as muscle cramps, aching, and clouding of thinking, are subjective and must be reported by patients, weakness and thermoregulatory symptoms may be amenable to objective measurement and warrant investigation as candidate biomarkers in future sensor-based studies. As PD is not primarily characterized by loss of muscle strength, weakness likely reflects physical exhaustion with patients often endorsing heavy-limbed sensations and weariness that make initiating tasks difficult. It may be related to reduced motor vigor or alterations in implicit motivation [14] which can be measured on behavioral and incentivized motor tasks [15,16,17]. However, self-reported weakness may overlap with bradykinesia, fatigue, or reduced motor vigor, and no sensor data were collected in this study. Its temporal association with dosing should therefore be regarded as a hypothesis for future wearable biomarker studies rather than evidence of an established biomarker. Wearable methods for quantifying thermoregulatory dysfunction in PD are still in the research stage but hold promise [18,19]. Potential biomarker candidates also lie in the Fluctuating–Heterogeneous quadrant, as we have noted the inherent non-uniformity of WO symptoms across individuals and the importance of tailored measures. However, it must be appreciated that the majority of symptoms in this quadrant are difficult to capture or quantify in real-time (e.g., anxiety, mood changes, abdominal discomfort, pain). With the evolution of large language models that power chatbots embedded in disease companions on mobile devices, some of these subjective symptoms may potentially be captured more accurately.

Some WO symptoms showed a close temporal relationship to dopaminergic dosing. In Cochran’s Q analysis, five symptoms varied significantly across the −2 to +2 h peri-dose window (Figure 3). Importantly, Cochran’s Q tests whether endorsement rates differ across bins; it does not, by itself, specify a single directional shape.

For experience of hot or cold (Q = 7.83, *p* = 0.024), the peri-dose profile peaked at dose time rather than rising monotonically before the dose: endorsement dipped slightly from −2 to −1 h, reached a maximum at 0 h, then fell sharply at +1 h before a partial rebound at +2 h. This still indicates dose-locked thermoregulatory fluctuation and is consistent with evidence linking thermoregulatory dysfunction in PD to dopaminergic tone [20,21], but the waveform is more consistent with a peri-dose peak than with a simple pre-dose OFF/post-dose ON pattern. We did not observe significant peri-dose modulation for sweating.

Abdominal discomfort was also significant (Q = 9.71, *p* = 0.044) but followed a different trajectory: highest at −2 h, declining toward dose time, with a transient rise at +1 h and 0% at +2 h—an inverted pattern relative to tremor, weakness, and muscle cramping, which peaked at 0 h. Given its low prevalence and sparse endorsements, this autonomic signal should be interpreted cautiously and is a weaker biomarker candidate than hot/cold or the more prevalent motor/sensory symptoms.

The sensation of weakness also showed significant peri-dose modulation and was moderately prevalent. Among motor symptoms, tremor and muscle cramping were significant; slowness of movement showed only a marginal trend (*p* = 0.051).

General stiffness did not show significant peri-dose modulation, possibly reflecting its more stable, trait-like expression (Stable–Uniform quadrant) rather than acute fluctuation with each dose cycle [22]. Residual heterogeneity in these dose-locked profiles may also reflect concomitant dopaminergic therapies (COMT inhibitors, MAO-B inhibitors, or dopamine agonists) used by a minority of participants, which were not modelled in stratified sensitivity analyses because of limited subgroup size.

The search for an ideal biomarker for WO thus converges on tremor, weakness, and experience of hot or cold as candidates for future sensor-based monitoring. Sweating, although measurable with wearables, did not show significant peri-dose modulation in this cohort.

### 4.3. Day-to-Day Consistency of WO Symptoms

Day-to-day symptom patterns were stable across most symptoms except panic attacks, pain and abdominal discomfort in Spearman analysis. This was supported by LME analysis on WOQ-19 total score showing no significant interactions with or main effects of study day on symptoms. The strong Day 2–Day 3 correlation of the overall WOQ-19 burden (rho = 0.682) and of most individual symptoms suggests that a few days of EMA may capture a consistent individual symptom profile. However, a correlation between two days does not by itself establish test–retest reliability, and longer monitoring periods are needed to confirm this.

### 4.4. Dissociation Between EMA Burden and Clinical Severity

The absence of significant correlations between mean WOQ-19 burden and conventional clinical measures should be interpreted cautiously given the small sample and wide confidence intervals (Table 2). Non-significant correlations do not demonstrate a true dissociation. Nonetheless, the pattern is consistent with EMA and clinic-based scales capturing complementary aspects of PD: brief visit ratings quantify capacity/severity at a single time point, whereas multi-day hourly EMA quantifies fluctuating lived burden. These modalities are best viewed as complementary rather than interchangeable.

### 4.5. Digital EMA as a Research and Clinical Tool

Our 91.9% completion rate over 72 h, with a mean reporting delay of approximately 2 min, demonstrates the feasibility of high-frequency EMA in community-dwelling adults with PD. This compares favourably to existing PD digital diary studies and suggests that well-supported hourly EMA is acceptable and achievable in this population. The hourly resolution also enabled us to detect the peri-dose symptom dynamic. These findings support the utility of high-frequency digital symptom capture as a research tool and as a component of future remote patient monitoring platforms for WO management.

### 4.6. Limitations

This study has several limitations. Firstly, the WOQ-19 is a binary symptom checklist that does not capture symptom severity; the presence of a symptom at any intensity is treated equally, making it difficult to implement treatment changes. While comprehensive, it still misses symptoms such as dysphagia or dyspnea; our digital version did not allow participants to enter symptoms beyond the listed ones. In addition, symptoms in the questionnaire are not well operationalized, and users may interpret them differently. It can be challenging for PwP to distinguish their symptoms because many aspects overlap (e.g., weakness can easily be mistaken for slowness). The WOQ-19 was validated as a retrospective instrument, and the psychometric validity of its repeated hourly administration has not been established; frequent administration may introduce participant fatigue or response habituation, and its short-interval test–retest reliability is unknown. Secondly, hourly sampling, while high by the standards of existing literature, still misses intra-hour fluctuations in fast-changing symptoms such as tremor. The 12 h time window also means that nighttime and early morning symptoms will be missed. Thus, the full spectrum and granularity of WO are not captured. Thirdly, meal timing data were not collected, so meal-related sensitivity analyses were not possible; protein intake can reduce peak levodopa plasma concentrations by up to 29% and delay absorption by approximately 34 min [23], and therefore remains an unmeasured confounder of peri-dose fluctuations. Fourthly, the study population was highly selected, requiring frequent self-reported wearing-off, smartphone familiarity, English fluency, and relatively preserved cognitive function; in addition, 7 of 30 recruited participants were excluded because of insufficient questionnaire compliance. These selection factors may inflate the apparent feasibility of high-frequency EMA relative to less selected clinic populations and limit generalizability of adherence estimates and symptom profiles. Comparisons between included and excluded participants (Supplementary Table S1) showed broadly similar disease duration and motor severity, with higher SCOPA-AUT scores in the excluded group as noted above. For Cochran’s Q, fixed ±2 h peri-dose windows were not adjusted for short inter-dose intervals; adjacent dosing cycles could therefore overlap in clock time, which may blur dose-specific timing contrasts for participants with frequent dosing. In a sensitivity analysis restricted to the 72 h protocol window, peri-dose modulation remained significant for weakness and experience of hot or cold, but not for tremor, muscle cramping, or abdominal discomfort; these latter findings should therefore be considered exploratory. Lastly, although levodopa/carbidopa was the primary dopaminergic regimen for all participants, concomitant medications that affect levodopa pharmacokinetics and pharmacodynamics—including entacapone (*n* = 3), MAO-B inhibitors (rasagiline, *n* = 1; selegiline, *n* = 1), and rotigotine (*n* = 2); altogether 7/23 participants—were not incorporated into stratified peri-dose models. Given these small subgroup sizes, formal sensitivity analyses would have been underpowered; adjunct therapy may nonetheless contribute residual heterogeneity in dose-locked symptom profiles.

## 5. Conclusions

This study provides a comprehensive characterization of wearing-off symptom expression in PD across 72 h of community-based, high-frequency EMA. WO symptoms are profoundly heterogeneous across individuals, yet are largely consistent from day to day within individuals. The ICC-CV quadrant framework offers a clinically interpretable taxonomy of symptom types to help guide biomarker choice. Our study confirms that high-frequency self-report captures a distinct dimension of lived disease experience that complements clinic-based assessment. Five symptoms demonstrated significant peri-dose modulation; of these, weakness and experience of hot or cold are candidates for future wearable sensor-based studies of levodopa cycling; as no sensor data were collected here, this remains to be tested. These findings support individualized approaches to WO monitoring and establish a foundation for digital phenotyping and machine-learning-based WO prediction in real-world settings. The significant rate of data loss highlights the administrative challenges of high-frequency EMA which may improve with technological advances. Larger and longer studies will help to clarify the generalizability of our findings. At the same time, the administrative and adherence demands of hourly EMA remain non-trivial: seven of thirty recruited participants did not meet the pre-specified compliance threshold, underscoring that high completion rates in the analyzed cohort should be interpreted in light of this selection.

## Figures and Tables

**Table 1:** Demographic and clinical characteristics of the analyzed cohort (*n* = 23).

Characteristic	Value
Age, years, mean ± SD	66.26 ± 8.47
Female, *n* (%)	10 (43.5%)
Disease duration, years, mean ± SD	9.61 ± 5.48
Hoehn & Yahr stage, *n* (%)	
Stage 1	6 (26.1%)
Stage 2	12 (52.2%)
Stage 3	5 (21.7%)
Hoehn & Yahr stage, median	2.0
MDS-UPDRS-III, mean ± SD	24.70 ± 11.57
MoCA, mean ± SD	26.87 ± 2.40
BDI-II, mean ± SD	8.96 ± 7.88
BAI, mean ± SD	11.52 ± 6.33
SCOPA-AUT, mean ± SD	12.18 ± 2.87
Self-reported WO episodes/day, mean ± SD	2.87 ± 1.42
Self-reported WO episodes/day, median (IQR)	2.5 (2–3)
Levodopa/carbidopa, *n* (%)	23 (100%)
Concomitant entacapone, *n*	3
Concomitant MAO-B inhibitor, *n* (rasagiline/selegiline)	2 (1/1)
Concomitant dopamine agonist (rotigotine), *n*	2
Any COMT/MAO-B/DA adjunct, *n* (%)	7 (30.4%)

Abbreviations: MDS-UPDRS-III, Movement Disorders Society–Unified Parkinson’s Disease Rating Scale Part III; MoCA, Montreal Cognitive Assessment; BDI-II, Beck Depression Inventory-II; BAI, Beck Anxiety Inventory; SCOPA-AUT, Scale for Outcomes in Parkinson’s Disease–Autonomic; WO, wearing-off; COMT, catechol-O-methyltransferase; MAO-B, monoamine oxidase B; DA, dopamine agonist.

**Table 2:** Spearman correlations between mean WOQ-19 total score and clinical measures; 95% confidence intervals were obtained by percentile bootstrap (10,000 resamples).

Clinical Measure	*n*	Spearman Rho	95% CI	*p*
MDS-UPDRS-III motor severity	23	−0.200	−0.604 to 0.240	0.361
Disease duration (years)	23	−0.248	−0.581 to 0.199	0.255
SCOPA-AUT	22	+0.097	−0.342 to 0.513	0.667
BAI score	23	+0.264	−0.115 to 0.591	0.224
Hoehn & Yahr stage	23	+0.155	−0.302 to 0.563	0.480

Abbreviations: WOQ-19, Wearing-Off Questionnaire-19; MDS-UPDRS-III, Movement Disorders Society–Unified Parkinson’s Disease Rating Scale Part III; SCOPA-AUT, Scale for Outcomes in Parkinson’s Disease–Autonomic; BAI, Beck Anxiety Inventory. SCOPA-AUT *n* = 22 due to one missing value.

## Data Availability

The datasets generated during and/or analyzed during the current study are not publicly available due to local personal data protection laws but are available from the corresponding author on reasonable request.

## Supplementary Materials

The following supporting information can be downloaded at https://www.mdpi.com/article/10.3390/jpm16100518/s1. Table S1. Comparison of demographic and clinical characteristics between included (*n* = 23) and excluded (*n* = 7) participants. Figure S1. Symptom prevalence across participants. For each symptom, prevalence is the mean frequency of hourly endorsement across all participants. Figure S2. Dose-locked symptom trajectory normalized to the inter-dose interval (IDI). Left: individual participant trajectories (grey) and cohort mean ± SEM (blue) of WOQ-19 total score, plotted against time since dose and expressed as a percentage of each dose cycle (0% = dose taken; 100% = next dose). Right: number of participants contributing to each bin, confirming stable coverage across the full dose cycle. Figure S3. Diurnal symptom burden. Mean (±SEM) WOQ-19 total score per hour of day for Day 2 (blue) and Day 3 (red), the two full monitoring days. Day 1 (baseline-visit day) and Day 4 were partial days and are not shown.
