# Supplementary material for: Heterogeneous Patterns of Wearing-Off in Parkinson’s Disease
Source: J Pers Med. 2026 Oct 3;16(10):518. doi: 10.3390/jpm16100518 (PMC13705877; doi:10.3390/jpm16100518)
Supplement: Supplementary file 1 [file jpm-16-00518-s001.zip › jpm-4518245-supplementary.pdf]

Supplementary Material

Statistical Analysis

For the construction of dose-locked symptom trajectories, to avoid survivorship bias (whereby participants with shorter inter-dose intervals (often those with higher symptom burden) contribute data only to early time bins) τ was normalized by each dose's inter-dose interval (IDI), expressing timing as a percentage of the dose cycle (0% = dose taken; 100% = next dose). Only within-day consecutive dose pairs were included. WOQ-19 total scores were averaged within 10 equal bins (deciles) of the normalized cycle, first within participant and then across participants to give equal weight to each individual regardless of dosing frequency.

For the Cochran's Q analysis, a binary dose-event × time-bin matrix was constructed for each symptom, including only those dose events for which a survey was present in each of the five hourly bins (−2, −1, 0, +1, +2 h relative to the dose); the survey closest to each bin centre was selected. A null distribution was generated across 1,000 permutations by shuffling the bin-label assignments within each participant's block of dose events, preserving within-person symptom rates while randomizing timing. Empirical p-values were computed as the fraction of permuted Q statistics equal to or exceeding the observed Q. Symptoms reaching $p < 0.05$ underwent McNemar post-hoc pairwise comparison against the 0-hour bin to identify the time bins driving significance.

Supplementary Table S1. Comparison of demographic and clinical characteristics between included (n = 23) and excluded (n = 7) participants.

Characteristic	Included (n=23)	Excluded (n=7)	p-value
Age (years)	66.26 ± 8.47	70.14 ± 7.15	0.258
Female, n (%)	10 (43.5%)	2 (28.6%)	0.669
Disease duration (years)	9.61 ± 5.48	8.67 ± 5.12	0.844
MoCA	26.87 ± 2.40	26.86 ± 1.95	0.803
BDI-II	8.96 ± 7.88	8.14 ± 5.64	0.805
BAI	11.52 ± 6.33	11.00 ± 8.94	0.694
SCOPA-AUT	12.18 ± 2.87	16.43 ± 4.61	0.018
UPDRS-III	24.70 ± 11.57	27.43 ± 9.43	0.572
Self-reported WO episodes/day (midpoint of range)	2.87 ± 1.42	2.50 ± 0.58	1.000
Hoehn & Yahr stage	1.96 ± 0.71	1.86 ± 0.38	0.780
Survey compliance (%)	91.86 ± 6.31	35.9–71.8	<0.001

Continuous variables are reported as mean ± SD and compared using the Mann–Whitney U test; sex was compared using Fisher's exact test. Disease duration is years from diagnosis to study start. Self-reported WO episodes/day were recorded as ranges and coded as the range midpoint. †Included group n = 22 (one missing value). Compliance = valid questionnaires within each participant's 72-hour protocol window ÷ 39 expected timepoints × 100%; excluded participants are shown as mean ± SD (range). Participants

with < 75% compliance were excluded as pre-specified, so the compliance difference is significant by design.

Supplementary Figure S1. Symptom prevalence across participants

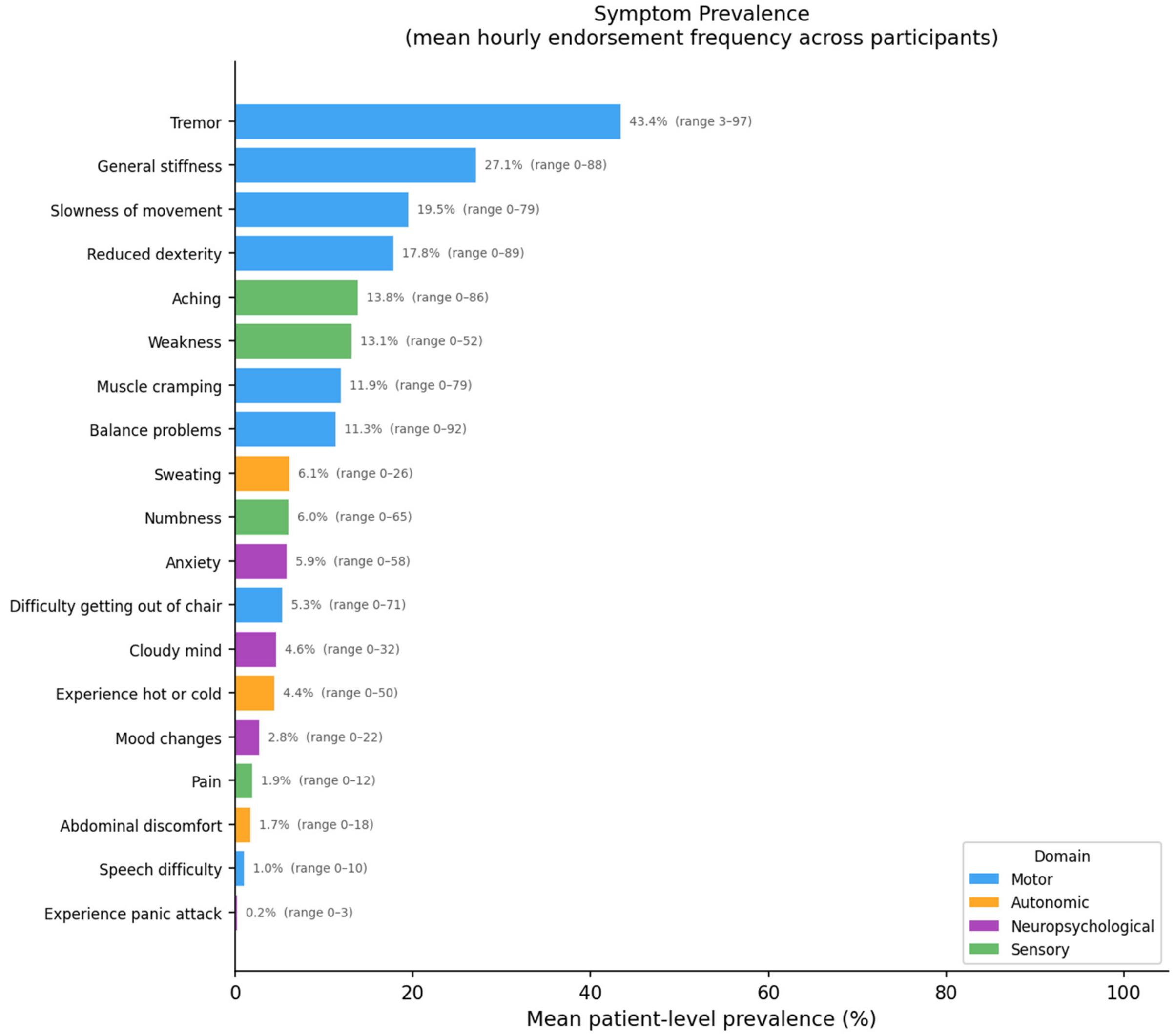

For each symptom, prevalence is the mean frequency of hourly endorsement across all participants.

Supplementary Figure S2. Dose-locked symptom trajectory normalized to the inter-dose interval (IDI)

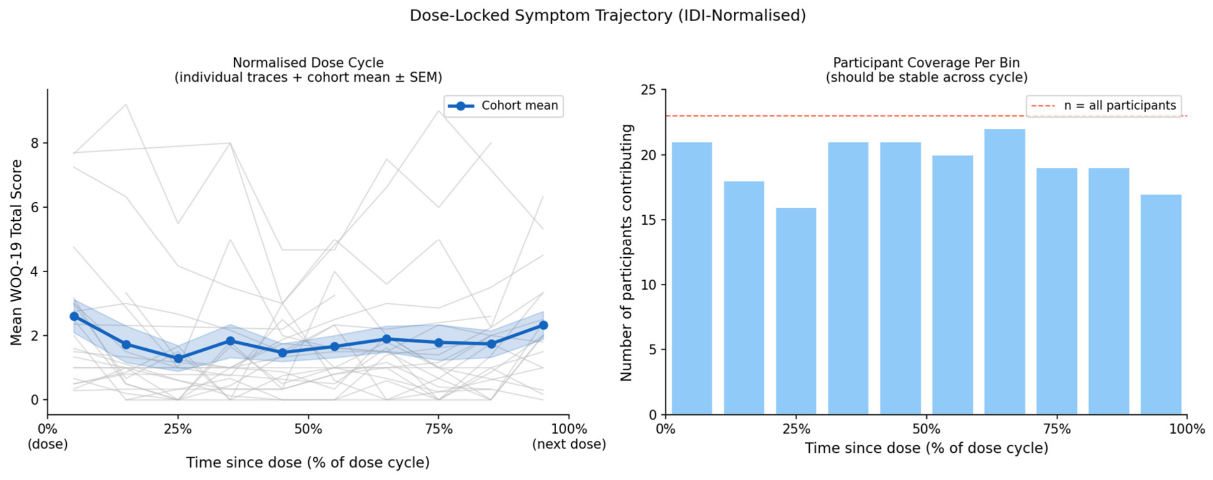

Left: Individual participant trajectories (grey) and cohort mean ± SEM (blue) of WOQ-19 total score were plotted against time since dose, expressed as a percentage of each dose cycle (0% = dose taken; 100% = next dose). Only within-day consecutive dose pairs were included (n = 23 participants; 714 survey–dose observations). To avoid survivorship bias from participants with shorter inter-dose intervals, τ was normalized by each dose's IDI before averaging. The cohort mean shows the expected pharmacodynamic pattern: highest burden at dose time (~0%), declining to a minimum around 25–45% of the cycle as levodopa takes effect, then rising again toward the next dose. Right: Number of participants contributing to each bin, confirming stable coverage across the full dose cycle.

Supplementary Figure S3. Diurnal symptom burden

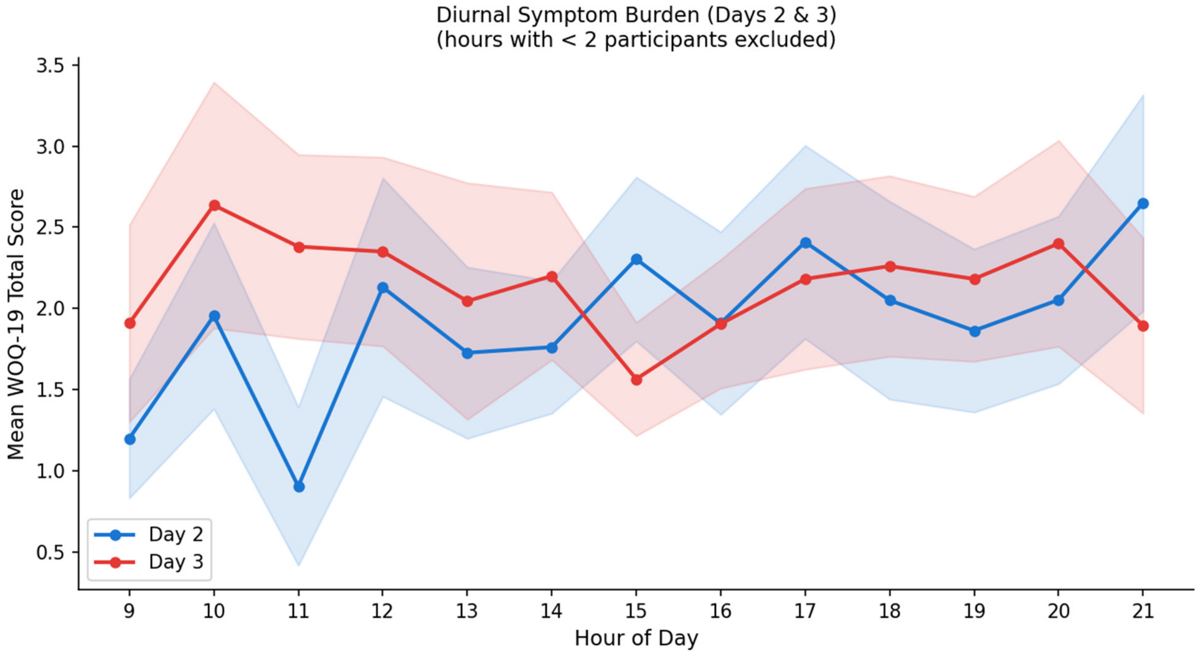

Mean (±SEM) WOQ-19 total score per hour of day were plotted for Day 2 (blue) and Day 3 (red). A non-significant trend toward diurnal worsening was observed (LME, $\beta = 0.031$ per hour, $p = 0.117$). Hours with fewer than 2 participants contributing data are excluded. Day-to-day profiles were consistent across both full study days.
